# A comparative assessment of coronary artery calcification on chest CT scans of patients referred to a cardio-oncology clinic

**DOI:** 10.1186/s40959-016-0017-z

**Published:** 2016-10-06

**Authors:** Alison M. Brann, Charlotte J. Bai, John F. Hibbeln, Kim A. Williams, Tochi M. Okwuosa

**Affiliations:** 1 Division of Cardiology, Rush University Medical Center, 1717 W. Congress Pkwy, Chicago, IL 60612 USA; 2 Department of Radiology, Loyola University Medical Center, 2160 South First Ave., Maywood, IL 60153 USA; 3 Cardio-Oncology Services, Rush University Medical Center, 1717 West Congress Parkway, Kellogg Bldg, Suite 320, Chicago, IL 60612 USA

**Keywords:** Cardio-oncology, Coronary artery calcium, Chest CT

## Abstract

**Background:**

The presence and burden of coronary artery calcium (CAC) is a strong predictor of cardiovascular events. In an effort to gain insight into the utility of CAC for coronary artery disease (CAD) screening in cancer patients with heart disease, we sought to determine the presence and burden of CAC detected on routine chest CT in patients referred to a cardio-oncology clinic, comparing them to a conventional cardiology clinic with the general population as controls.

**Methods:**

Patients from the cardio-oncology clinic, general cardiology clinic, and the general clinic population at Rush University Medical Center who had a chest CT as part of their previous treatment were identified. Each CT scan was evaluated for presence, extent, and severity of CAC by 3 independent readers.

**Results:**

In multivariate analysis, when compared with cardio-oncology clinic, CAC was more prevalent in the CT scans of cardiology patients (*p* = 0.04), but not the general clinic population (*p* = 0.5); CAC extent (*p* = 0.05) and severity (*p* = 0.05) was significantly higher in the cardiology patients but the extent (*p* = 0.05) and severity (*p* = 0.92) was similar in the general clinic population.

**Conclusion:**

Despite being matched by age and sex, controlling for other major cardiovascular risk factors, patients referred to our cardio-oncology clinic had similar and less prevalent/severe CAC burden compared with the general population and conventional cardiology clinics respectively. Whether this translates to less utility of CAC for CAD screening, or to less overall coronary events in a cardio-oncology clinic, is of interest.

## Background

Advancements in oncological treatments over the past few decades, including chemotherapeutics, radiation therapy and anti-cancer signaling inhibitors, have greatly improved the survival outcome of patients with cancer. Unfortunately, many of these treatment options carry significant risk of cardiovascular complications, especially among patients with underlying cardiovascular disease (CVD) [1]. Additionally, survivors of several types of cancers have been shown to have an increased risk of cardiovascular complications [2, 3]. In recent years, the field of cardio-oncology has emerged in an effort to optimize outcomes in cancer patients with heart disease. Cardio-oncologists work both to treat cardiovascular complications that develop following cancer treatment, and also to identify patients who are at risk of developing cardiovascular issues in order to help safely guide cancer treatment and possibly limit morbidity and mortality associated with cardiotoxic therapies [4].

Assessing the presence and burden of coronary artery calcium (CAC) is an attractive option for assessing cardiac risk in a cardio-oncology clinic as cancer patients often undergo routine screening chest CT scans in which CAC is readily detected. To this end, there is very strong data showing that higher CAC burden (assessed by Agatston scores measured by gated CT scans) carries greater risk for CHD events [5, 6]. The presence and degree of CAC has been shown to reflect the overall atherosclerotic burden and is correlated with the presence of coronary stenosis [7]. In asymptomatic patients, CAC-based models have been shown to predict future cardiovascular events [8, 9] and mortality [10], independent of the classic cardiovascular risk factors. Inclusion of the CAC score into cardiac risk stratification has been shown to predict coronary events beyond the traditional Framingham risk factors across all racial groups [11] and particularly in intermediate risk groups, in whom clinical decision-making is most uncertain [12]. Furthermore, the absence of CAC is associated with higher survival and effectively allows a patient to remain classified as low risk for a period of 15 years [13].

It is notable that CAC is identifiable on non-cardiac chest CT, such as those used for screening and staging purposes in cancer patients. In fact, CAC identified on a routine diagnostic chest CT scan has been found to predict cardiovascular events with an adjusted risk of CVD events that was almost 4 times higher among patients with severe CAC in one study [14]. In one study of 60 patients, the correlation coefficients between gated CT for Agatston CAC scoring and non-gated CT for routine chest examinations were 0.95, 0.97 and 0.98 for volume, mass and Agatston values, respectively [15]. Findings were similar in the National Lung Screening Trial (NLST) of 1575 CT scans [16]. In another study, the sensitivity and specificity of routine chest CT scanning for detecting positive CAC scores were 96.4 % and 100 %, respectively [17]. Nonetheless, the use of CAC in assessing cardiac risk in cancer patients having undergone cancer therapy, is unknown.

In our study, we aimed to compare the presence and burden of CAC seen on routine chest CT scans in patients referred to a cardio-oncology clinic. We postulated that information gleaned from our study would provide some insight into the burden of CAD – and therefore projected burden of cardiovascular events based on CAC – in our post treatment cancer patients with heart disease, compared with a regular cardiology clinic, and using the general population as controls. This would also provide some initial data on the utility of CAC – performed on surveillance chest CT scans – for coronary artery disease (CAD) screening in cancer patients with heart disease. Furthermore, if these chest CT scans obtained for other (non-cardiac) reasons are found to be useful screening tools in this population, one could possibly avoid radiation exposure associated with other screening modalities, such as stress myocardial perfusion imaging, coronary CT angiography, and dedicated CT screening for CAC by the Agatston scoring system.

## Methods

The cases for the current study were identified from referrals made to the cardio-oncology clinic at the Rush University Medical Center in Chicago, IL. Patients aged 20–79 years with a history of malignancy who visited the cardio-oncology clinic in the time period of January to June 2014, and who had a chest CT performed as part of their cancer diagnostic/surveillance regimen, were eligible for the study. In order to obtain a comprehensive view of the cardio-oncology clinic population, we included all types of cancer diagnoses and did not exclude patients with known CAD or other heart disease. Our case population was then matched by age, sex and date of chest CT to patients with no cancer history who visited the conventional cardiology clinic at Rush, as well as to a second group of controls matched by age, sex and date of chest CT with no cancer history selected from the general clinic population of patients at Rush. The control groups consisted of both inpatients and outpatients who underwent chest CT imaging not specifically performed for oncologic staging or heart disease. The study was approved by the Rush University Institutional Review Board, and informed consent was waived due to the retrospective nature of the study.

Information about each patient was collected through a retrospective review of the electronic medical record (EMR). Information regarding patient demographics, cardiac risk factors, medication use and known history of coronary artery disease, myocardial infarction, coronary artery bypass graft, stent placement and end-stage renal disease were recorded. For patients with cancer, the date of diagnosis, type of cancer, and history of treatment with radiation and/or chemotherapy were also recorded.

Each chest CT was evaluated for presence, extent, and severity of CAC by three, experienced readers who were blinded to clinical information and the purpose of the study, including a board certified radiologist, and two cardiologists: with board certifications in cardiac CT. In an effort to mimic daily clinical practice, we chose to include CT scans from a variety of different CT imaging protocols. The CT imaging technique and protocols remained the same at our institution throughout the period of the study. All CT scanners were multi-detector spiral CT (MDCT) scanners with 16, 64 or higher order row systems with similar in-plane resolution. Four separate models of CT scanners were used including a Philips Brilliance 64 (Eindhoven, Netherlands), GE Bright Speed Profile 16 (Waukesha, Wisconsin), Siemens Definition AS (Erlangen, Germany), and Siemens Somatom Definition Flash (Erlangen, Germany). All images were obtained with single breath hold technique, using thin section acquisition with 3 mm slice thickness or less, consistent with the varied clinical reasons for the scan. A 120 keV tube voltage was applied in the majority of cases, although a range of 80 keV to 140 keV was used (with those platforms equipped with keV dose modulation). Image acquisition was obtained with optimization of radiation dose, utilizing angle dependent dose modulation. Image reconstruction was performed utilizing medium and higher frequency reconstruction kernels with respective mediastinal and lung windows. Images were interpreted with the mediastinal reconstructions. None of the studies were electrocardiographically prospectively triggered or retrospectively gated.

The chest CT scans were reviewed for the presence, extent, and severity of CAC within the left main coronary artery (LMCA), left anterior descending (LAD), left circumflex coronary artery (LCX) and right coronary artery (RCA) using a previously published semi-quantitative visual grading scale [18]. Each vessel was given a score of 0 to 3 for extent defined as 0 = no foci; 1 = focal, a single focus; 2 = moderate, >1 focus; or diffuse = 3, foci in proximal, mid-, and distal segments. Each vessel was also given a score of 0 to 3 for severity defined as 0 = no foci, 1 = mild, 2 = moderate and 3 = severe. The scores for severity of each vessel were summed to assign a total CAC severity score for each patient (0–12).

Baseline characteristics were compared in univariate analysis. Logistic regression was performed to compare differences in CAC presence between groups while linear regression was used to assess the relationship between CAC severity and all 3 groups, adjusting for race, body mass index (BMI), cardiovascular risk factors, and use of cardiac medications. A time-dependent variable (from cancer therapy to date of CT scan) was also introduced into the models as a covariate. Furthermore, inter-observer variability between readers was assessed using linear regression. Data are presented as mean value ± 1 SD, and *P* value < 0.05 is considered statistically significant.

## Results

A total of 83 patients from the cardio-oncology clinic met the inclusion criteria and were included in this study. The mean age was 60 years (range 23–69) and 47 % were men (Table 1). The most common types of cancer represented were breast cancer (27.7 %), lymphoma (15.7 %), lung cancer (13.3 %) and gynecologic cancers (13.3 %) – Table 2. A history of treatment with chemotherapy was present in 76 of the 83 patients (91.6 %). A total of 33 (39.8 %) patients received radiation therapy, out of which 24 received chest wall radiation – 7 of these patients had lung cancer, 5 with lymphoma, 6 with left-sided breast cancer, and 6 with right-sided breast cancer. The mean time from radiation therapy to time of chest CT for patients receiving radiation to the left side of the chest was 1648 days (1327 days for left-sided breast cancer, 4145 days for lymphoma and 184 days for patients with lung cancer). Unfortunately, data regarding the dose of radiation received by each patient were not available. A total of 4 patients, out of the 18 patients who received left chest radiation, received radiation therapy prior to the institution of modern, cardiac dose limiting techniques. The most common reasons for referral to the cardio-oncology clinic were cardiomyopathy (24.1 %), management of cardiovascular risk factors during chemotherapy (19.2 %), and hypertension (10.8 %). The average amount of time from the date of cancer diagnosis to the date of the chest CT used was 4.9 years (median 1.5 years). Out of the 83 cardio-oncology patients, 79 were undergoing active therapy at the time of the chest CT scan and 4 were in remission (average 16.25 years since last cancer treatment). Of those undergoing active therapy, 7 were being treated for a second malignancy, after having completed treatment for a separate malignancy on average 12.4 years prior. The baseline characteristics and cardiovascular risk factors present in each group are detailed in Table 1. In univariate analysis, despite matching for age and sex at baseline, the conventional cardiology group had higher rates of cardiovascular disease and risk factors, and were less likely to be white than the cardio-oncology clinic and general clinic population group.

**Table 1:** Baseline characteristics

General Characteristics	Cardio-Onc Clinic	Cardiology Clinic	General clinic population	*P*-value
Total Number of patients	83	83	83	
Number of men	35	41	41	0.60
Average age	60.12	60.52	59.69	0.91
Race	White = 49	White = 29	White = 34	0.03
	Black = 23	Black = 39	Black = 35	
	Hispanic = 11	Hispanic = 12	Hispanic = 12	
	Asian = 0	Asian = 2	Asian = 1	
	Other = 0	Other = 1	Other = 1	
Smoking Status	Never = 40	Never = 40	Never = 48	0.05
	Former = 35	Former = 29	Former = 18	
	Current = 8	Current = 14	Current = 17	
No. with *any* known CAD, MI, CABG *or* stent	18	44	6	<0.01
No. with CKD or ESRD	6	31	12	<0.01
No. Family history of MI	37	31	22	0.05
Average systolic BP	127.1 +/- 19.5	126.6 +/- 21.5	131.4 +/- 21.8	0.27
Average diastolic BP	70 +/- 12.2	71 +/- 12.4	77.2 +/- 13.5	<0.01
Average BMI	27.7 +/- 5.6	29.7 +/- 7.2	27.5 +/- 9.7	0.14
No. with HTN or HTN treatment	48	72	50	<0.01
No. with DM or DM treatment	13	29	21	0.02
No. with dyslipidemia or lipid treatment	35	51	27	<0.01
No. on aspirin	19	52	20	<0.01
No. on beta blockers	27	53	18	<0.01
No. on ACEI or ARB	35	33	22	0.08
No. on nitrates	3	4	1	0.4

*ACEI* Angiotensin-converting enzyme inhibitor, *ARB* Angiotensin receptor blocker, *BMI* Body mass index, *BP* Blood pressure, *CABG* Coronary artery bypass grafting, *CAD* Coronary artery disease, *CKD* Chronic kidney disease, *DM* Diabetes mellitus, *ESRD* End stage renal disease, *HTN* Hypertension, *MI* Myocardial infarction

**Table 2:** Cancer diagnoses/treatment in the cardio-oncology population

Cancer Diagnosis/Treatment	No. patients	%
Breast Cancer	23	27.71 %
Lymphoma	13	15.66 %
Lung Cancer	11	13.25 %
Gynecologic Cancers	11	13.25 %
Multiple Myeloma/Myelodysplastic Syndromes	10	12.05 %
Leukemia	8	9.63 %
Head and Neck Cancer	2	2.4 %
Sarcoma	2	2.4 %
Colorectal Cancer	1	1.2 %
Renal Cell Cancer	1	1.2 %
Neuroendocrine Tumor	1	1.2 %
Chemotherapy alone	46	55.4 %
Chemotherapy plus radiation therapy	30	36.1 %
Radiation alone	3	3.6 %
Left sided radiation for breast cancer	6	7.2 %
Right-sided radiation for breast cancer	6	7.2 %
Chest radiation for lymphoma	5	6.0 %
Chest radiation for lung cancer	7	8.4 %

A total of 249 chest CT scans were each reviewed separately by three readers. The findings of the three readers demonstrated excellent agreement in the total CAC severity score, with inter-observer correlation coefficients of 0.92, 0.93 and 0.97 for each 2 of 3 readers (*p* < 0.01).

CAC was found to be present in 110 of the 249 total patients included in this study: 35 patients (42 %) from the cardio-oncology clinic, 40 patients (48 %) from conventional cardiology and 35 patients (42 %) from the general clinic population. In multivariate analysis (Table 3), the presence of CAC was significantly lower in the cardio-oncology clinic compared with the conventional cardiology clinic (OR 0.334; 95 % CI: 0.12, 0.96; *p* = 0.04), while the cardio-oncology and general clinic population were found to have a similar prevalence (OR 1.34; 95 % CI: 0.55, 3.4; *p* = 0.5). The overall CAC severity score, of a possible 996, was 254 in the cardio-oncology clinic, 363 in conventional cardiology, and 167 in the general clinic population. In multivariate analysis, the total severity of CAC was significantly lower in the cardio-oncology clinic compared with the conventional cardiology clinic (OR 0.33; 95 % CI: 0.11, 1.00; *p* = 0.05). The total severity of CAC in the cardio-oncology and general clinic populations was not significantly different (OR 1.05; 95 % CI: 0.41, 2.72; *p* = 0.92). Analysis of the extent and severity of CAC in individual arteries, such as the LMCA and LAD, showed a similar pattern (Table 3). A comparison of the cardio-oncology patients who received whole chest or left sided chest radiation to the two control groups revealed similar results. These patients had higher levels of CAC than the general population and lower levels of CAC than the conventional cardiology clinic.

**Table 3:** Multivariate odds ratios and 95 % confidence intervals for CAC presence/extent compared with cardio-oncology

Total CAC Presence:		
Cardiology	0.334 (0.12, 0.96)	*p* = 0.04
General clinic population	1.34 (0.55, 3.4)	*p* = 0.5
Total CAC Severity:		
Cardiology	0.33 (0.11, 1.00)	*p* = 0.05
General clinic population	1.05 (0.41, 2.72)	*p* = 0.92
CAC Presence in Left Main Coronary Artery:		
Cardiology	0.29 (0.1, 0.83)	*p* = 0.02
General clinic population	0.47 (0.19, 1.2)	*p* = 0.16
CAC Severity in Left Main Coronary:		
Cardiology	0.19 (0.05, 0.7)	*P* = 0.013
General clinic population	0.16 (0.04, 0.61)	*p* = 0.92
CAC Extent in Left Anterior Descending:		
Cardiology	0.36 (0.13, 1.02)	*p* = 0.05
General clinic population	1.36 (0.55, 3.35)	*p* = 0.5
CAC Severity in Left Anterior Descending:		
Cardiology	0.39 (0.13–1.23)	*p* = 0.1
General clinic population	1.23 (0.44, 3.42)	*p* = 0.69

Adjusted by number with any known coronary artery disease, myocardial infarction, coronary artery bypass grafting, number with chronic kidney disease or end stage renal disease, family history of myocardial infarction, number with hypertension or hypertension treatment, number with diabetes mellitus or diabetes treatment, number with dyslipidemia or lipid treatment, number on aspirin, average systolic BP, average BMI, and age, sex, date of chest CT scan match

## Discussion

In an effort to understand the relative risk of cardiovascular events in cancer patients with cardiac concerns referred to a cardio-oncology clinic, this single-center retrospective observational study evaluated the prevalence and severity of CAC in these patients, in comparison to cardiac patients with no history of cancer referred to a regular cardiology clinic, and to non-cancer patients from the general clinic population. Even after matching for age and sex, controlling for cardiovascular risk factors including BMI, and time-dependent variable (from cancer treatment to date of CT scan), patients referred to the conventional cardiology clinic had higher prevalence, extent and severity of CAC compared with those referred to the cardio-oncology clinic. There was no significant difference in the burden of CAC in the cardio-oncology clinic compared to the general clinic population.

To our knowledge, this is the first study investigating the presence and burden of CAC seen on routine chest CT scans in a cardio-oncology clinic. The study included a wide range of cancer diagnoses and treatments, along with a variety of reasons for referral to the cardio-oncology clinic, mimicking that seen in clinical practice. A somewhat unexpected finding is that those referred to the cardio-oncology clinic had less CAC, despite the fact that about a quarter of them (18 patients) received left sided or whole chest radiation, which would be expected to increase the presence and severity of atherosclerosis [19], and therefore CAC detected on chest CT. A separate analysis comparing only those cardio-oncology patients who received left-sided or whole chest radiation revealed that these patients had higher levels of CAC than the general population, but lower levels of CAC than the conventional cardiology clinic. These findings could have been affected by the low number of patients that received left sided radiation therapy (*n* = 18; 6 patients with left sided breast cancer, 5 patients with lymphoma, and 7 patients with lung cancer), as well as the limited number of patients in the study overall. Data are limited on the prevalence of CAC post chest wall radiation [20], and one small study has suggested absence/low levels of CAC in patients referred for CT scanning after radiation therapy for breast cancer [21]. The possible lack of calcification in the radiation plaque may have something to do with known differences in the histology of atherosclerosis for the radiation-induced arterial plaque relative to the more traditional (non-radiation induced) plaque. While the histology of the traditional atherosclerosis involves more macrophage-driven atheromatous plaque, the process for radiation-induced atherosclerosis leads to fibrous plaque. Thus, despite its utility in predicting CHD and CVD events in the general population, further larger and more diverse studies are required to evaluate the utility of CAC screening for radiation-induced heart disease and cardiovascular events.

It is also possible that our results reflect a referral bias between the two clinics. The patients in the cardiology group had a significantly higher amount of baseline CAD. Patients who present to a cardio-oncology clinic are commonly referred for reasons other than CAD; such as cardiomyopathy, hypertension and arrhythmias. Additionally, cardio-oncology clinics are comprised of non-invasive cardiologists, compared with the conventional cardiology clinic made up of both general and interventional cardiologists - translating to more severe CAD. It must be noted that patients with a history of cancer were excluded from the conventional cardiology group in this study. As such, cancer patients with CAD requiring treatment by an interventional cardiologist were not included in this study. Further investigation, in a more randomized fashion, is needed before conclusions can be made regarding levels of CAC in patients with heart disease with and without cancer.

Previous research investigating associations between cancer and CAC are limited and have shown mixed results. A recent study utilizing the Multi-Ethnic Study of Atherosclerosis (MESA) cohort found that a diagnosis of cancer and its treatment was associated with an increased incidence of developing new CAC in both men and women, even after accounting for atherosclerotic risk factors [22]. This was the first study to investigate the longitudinal change of CAC with a cancer diagnosis, compared to the general population. A smaller, cross-sectional cohort study of 80 breast cancer patients, demonstrated significantly higher CAC scores prior to receiving radiation therapy, compared to controls from the MESA cohort. However, this difference was only significant in the age category 55–64 years [23]. Studies investigating the effect of cancer treatment on CAC scores have had mixed results [24, 25].

Our study differs from prior studies in several ways. The patients were selected from a cardio-oncology clinic and a wide range of cancer types and past treatments were included. Additionally, our comparison groups consisted of non-cancer patients selected from the same hospital, rather than historical controls or published cohort values. Lastly, we were able to use chest CT scans that had already been recorded as part of each patient’s past cancer workup.

Limitations of this study should be acknowledged. First, this was not a prospective, randomized study. The population studied was a small retrospective cohort from one single medical center and the small sample size may have limited the power of our study. Of note, prior CAC studies have been conducted with a smaller number of patients. Our inclusion criteria limited the number of patients at our hospital available for potential matches. Therefore, the groups are not perfectly homogenous according to race and sex, although the difference for sex was not statistically significant. We included all types of malignancies and prior anti-cancer treatment histories for which we did not adjust; although we adjusted for the higher rates of cardiac disease and risk factors in the general-cardiology group, the possibility of residual confounding remains. We included CT scans recorded for a variety of clinical indications with varying acquisition protocols. However, the scanners included used similar imaging techniques. Additionally, we demonstrated good concordance amongst our three readers, indicating that any variation in scan technique resulted in the same interpretation. The CT scans were ungated, so small foci of calcification could have been missed due to motion artifact from the heart. However, low dose ungated MDCT has been shown to be reliable for predicting the presence of CAC and assessment of Agatston score, with excellent correlation between gated and ungated MDCT [26]. Lastly, we used a simplified visual CAC scoring system; and while it is not validated, it has been shown to be reliable for predicting the presence of CAC and assessment of Agatston score [26]. Studies using simple ordinal scoring systems, similar to the system used in our study for the visualization of CAC on ungated-low dose CT scans recorded for screening and diagnostic purposes have shown a correlation between CAC severity and cardiovascular events and death [5, 27]. The high degree of concordance in CAC scoring amongst the reviewers despite differences in experience and CT scanners utilized demonstrates the ease and robustness of the employed semi-quantitative technique for its application in a variety of patient populations and general practice.

## Conclusion

Despite being closely matched by age and sex, and controlling for other major cardiovascular risk factors, patients referred to our cardio-oncology clinic had similar and less prevalent/severe CAC burden compared with the general population and conventional cardiology clinics, respectively. This was an unexpected finding given that a third of our cardio-oncology clinic population received radiation therapy known to be associated with long-term accelerated development of atherosclerosis. It is possible that differences in atherosclerosis plaque composition translate to the eventual CAC end-point in coronary vessels. Whether this translates to less utility of CAC for CAD screening (or to less overall coronary events) in a cardio-oncology clinic, is of interest. The cardio-oncology population receives multiple staging and surveillance chest CT scans, which could easily translate into a readily available coronary event risk prediction, if found to be a useful tool for this purpose. As such, more investigation into the utility of CAC screening – by dedicated or undedicated chest CT scans – for coronary or cardiovascular events, is warranted in this population.

## Abbreviations

ACEI: Angiotensin-converting enzyme inhibitor
ARB: Angiotensin receptor blocker
BMI: Body mass index
BP: Blood pressure
CABG: Coronary artery bypass grafting
CAC: Coronary artery calcium
CAD: Coronary artery disease
CKD: Chronic kidney disease
CT: Computerized tomography
CVD: Cardiovascular disease
DM: Diabetes mellitus
EMR: Electronic medical record
ESRD: End stage renal disease
HTN: Hypertension
LAD: Left anterior descending
LCX: Left circumflex coronary artery
LMCA: Left main coronary artery
MDCT: Multi-detector spiral CT
MESA: Multi-Ethnic Study of Atherosclerosis
MI: Myocardial infarction
RCA: Right coronary artery
